# Focal Task-Specific Dystonia Beyond M1: Network Adaptations and a Causal-Architecture Taxonomy of Dystonia

**DOI:** 10.3390/brainsci16090985

**Published:** 2026-09-17

**Authors:** Norman Lu, Rodrigo F. O. Pena

**Affiliations:** Department of Biological Sciences, Florida Atlantic University, Jupiter, FL 33458, USA; normankwlu@gmail.com

**Keywords:** focal task-specific dystonia, primary motor cortex, task-specific motor synergy, network adaptation, neuroplasticity, basal ganglia, cerebellum, symptom-threshold, below- or at-threshold retraining, causal architecture

## Abstract

**Highlights:**

**What are the main findings?**

- Repeated expression of a task-specific motor synergy with a proposed M1 excitation–inhibition imbalance is hypothesized to produce task-linked adaptations in striatal, cerebellar, somatosensory, and spinal circuits.
- A causal-architecture taxonomy distinguishes typical neuroplastic, atypical neuroplastic, and non-neuroplastic dystonias by the process proposed to dominate symptom generation and persistence.

**What are the implications of the main findings?**

- Temporal and intervention-based tests can distinguish an M1-primary sequence, defined by causal initiation within a task-relevant M1 TSMS, from basal ganglia-primary, cerebellar-primary, sensory-primary, concurrent, or distributed alternatives.
- Prospective studies should test whether BATR is most directly applicable to task-specific neuroplastic dystonias with an identifiable intact functional range and measurable symptom threshold.

**Abstract:**

Focal task-specific dystonia (FTSD) is associated with abnormalities across primary motor cortex (M1), basal ganglia, cerebellum, primary somatosensory cortex (S1), and spinal circuits, but their causal ordering is unresolved. Starting from the companion M1-centered framework, we ask whether repeated expression of a task-specific motor synergy (TSMS) with a proposed M1 excitation–inhibition imbalance could produce task-linked adaptations elsewhere in the motor system. We propose candidate mechanisms linking abnormal M1 output to reweighting of striatal dopamine signaling and direct- and indirect-pathway function, recalibration of cerebellar teaching and corrective output, reduced functional separability of S1 sensory populations, and use-dependent weakening of spinal reciprocal inhibition. Reported FTSD findings serve as empirical constraints; these intermediate cellular and circuit links remain hypotheses rather than established causal sequences. Extra-M1 changes could later reinforce or help maintain the dystonic state, compensate for it, or interact bidirectionally with the cortical abnormality. The M1-primary claim concerns causal initiation rather than anatomical exclusivity and predicts that task-specific abnormalities of M1 recruitment and rapid inhibitory control should precede or closely track the proposed extra-M1 adaptations. We also propose a provisional causal-architecture taxonomy distinguishing typical neuroplastic, atypical neuroplastic, and non-neuroplastic dystonias by the process hypothesized to dominate symptom generation and persistence. The TSMS framework, including the symptom-threshold, overreaching sequence, a proposed state of task-specific output limitation termed true weakness, and below- or at-threshold retraining (BATR), is advanced for FTSD rather than assumed to generalize unchanged across dystonias. Longitudinal, intervention-based, and experimental-model tests are outlined to distinguish the proposed sequence from basal ganglia-primary, cerebellar-primary, sensory-primary, concurrent, and distributed alternatives and to test whether retraining-related improvement follows the predicted physiological route.

## 1. Introduction

Focal task-specific dystonia (FTSD) is an isolated dystonia in which abnormal muscle contractions are elicited during a particular motor activity, such as writing, instrumental performance, phonation, or a sport-specific task, while most other movements remain relatively spared [1,2,3,4,5]. FTSD is associated with abnormalities across a distributed motor network that includes primary motor cortex (M1), basal ganglia, cerebellum, thalamus, somatosensory cortex, and spinal circuits [6,7,8]. These findings establish multilevel involvement but do not determine which abnormality initiates the disorder, which develops concurrently, or which later compensates for or reinforces it. Basal ganglia-primary, cerebellar-primary, sensory-primary, premotor- or associative-cortical-primary, and distributed-network accounts, therefore, remain viable [9,10]. The marked task specificity of FTSD, relative sparing of other movements, intensity-dependent symptom expression, and reports of improvement with carefully constrained retraining nevertheless motivate testing a task-specific cortical motor-generating hypothesis [11,12,13].

To address that possibility, the companion Hypothesis article, “Focal Task-Specific Dystonia: An M1-Centered TSMS Framework,” defined a task-specific motor synergy (TSMS) as a hypothesized task-dependent ensemble of pyramidal neurons and local inhibitory interneurons in M1 that contributes to a particular unidirectional movement component within a learned task [14]. The companion framework proposes that recurrent excitation within an affected TSMS becomes disproportionately strong relative to rapid parvalbumin (PV)-centered inhibitory control, producing a relative excitation–inhibition (E/I) imbalance and an excitatory-dominant dystonic configuration that is preferentially recruited as task demand increases. Reduced short-interval intracortical inhibition (SICI) and disturbed surround inhibition are compatible with a fast inhibitory deficit, but they do not identify a TSMS or directly establish PV-interneuron dysfunction; PV-centered involvement remains a mechanistic inference. The framework also proposes that altered movement coordination can reduce functional TSMS capacity, producing a proposed state of task-specific output limitation termed true weakness at higher attempted intensities, and that repeated overreaching beyond the retained capacity can bias plasticity toward excitation. The symptom-threshold denotes the highest task intensity, under a specified performance condition, at which overt dystonia remains absent. Below- or at-threshold retraining (BATR) is defined as systematic practice at or below the lower of this threshold and, where present, the true-weakness threshold, so that attempted intensity and realized output remain matched and overt dystonia is absent.

The present article takes the TSMS construct, proposed M1 E/I imbalance, symptom-threshold, true weakness, overreaching, and BATR as starting hypotheses rather than re-deriving them. It develops three distinct extensions: candidate routes by which repeated abnormal M1 output could reshape striatal, cerebellar, somatosensory, and spinal circuits; hypotheses about the reinforcing, maintaining, or compensatory roles those adaptations could later acquire and a provisional causal-architecture taxonomy of dystonia with testable implications for motor retraining. The central network-level proposition is that repeated expression of the dystonic synergy within an affected M1 TSMS could produce task-linked adaptations elsewhere in the motor system, some of which may subsequently feed back onto M1 and contribute to persistence.

In this article, M1-centered describes the organizational focus of the framework, whereas M1-primary denotes a specific claim about circuit-level causal initiation: the circuit abnormality hypothesized to initiate the dystonic state arises within a task-relevant M1 TSMS. The term does not imply that susceptibility factors cannot precede that circuit abnormality, that FTSD is anatomically confined to M1, that M1 is the only abnormal node, or that extra-M1 abnormalities cannot later become necessary for persistence. We use initiating mechanism for an abnormality that precedes and contributes to initial formation of the dystonic phenotype; maintaining mechanism for one that remains necessary for, or materially contributes to, continued symptom expression after onset; reinforcing mechanism for one that increases the stability, severity, or probability of symptom expression without necessarily being required for initiation and compensatory mechanism for a response that opposes or limits the abnormal state, even if incompletely. In a concurrent or distributed account, abnormalities arise across multiple motor-network nodes without demonstrated or reproducible primacy of a single node. The terms secondary and downstream refer only to proposed temporal and causal ordering and do not imply that an abnormality is epiphenomenal, clinically unimportant, or uniformly maladaptive.

This Hypothesis article presents a hypothesis-driven, non-systematic synthesis rather than an exhaustive review or formal evidence-grading exercise. It focuses on the striatal, cerebellar, somatosensory, and spinal findings most directly relevant to the network-adaptation question, together with experimental literature used to assess the biological plausibility of the proposed intermediate steps. Reported clinical and systems-level observations anchor the analysis, whereas the links from repeated abnormal M1 output to those observations remain mechanistic hypotheses. Conflicting or methodologically heterogeneous findings are addressed where they materially constrain a proposed mechanism. Compatibility with the M1-centered account is not treated as evidence that M1 causal primacy has been established.

The TSMS, symptom-threshold, true weakness, overreaching, and BATR constructs imported from the companion framework, together with the extra-M1 adaptation mechanisms developed here, are advanced specifically for FTSD phenotypes with an identifiable task-bound performance state. Their extension to non-task-specific, lesion-associated, or molecularly defined dystonias is not assumed. The broader causal-architecture taxonomy generalizes only the proposition that disorders sharing dystonic phenomenology may differ in their dominant causal architecture, dependence on motor plasticity, and predicted response to retraining. It is a provisional interpretive framework rather than an individual-level diagnostic algorithm. Section 2 develops the four candidate extra-M1 adaptation pathways and their discriminating predictions. Section 3 examines the causal-architecture taxonomy, evidentiary limitations, competing causal sequences, and tests that could support, weaken, or require revision of the proposed framework.

## 2. Network Adaptations Beyond M1

In the sections that follow, reported basal ganglia, cerebellar, somatosensory, and spinal findings in FTSD are treated as empirical constraints on candidate mechanisms of network adaptation. Each subsection is organized in three stages: the reported phenotype and limits of its measurement; a conditional mechanism that could connect repeated abnormal M1 output to that phenotype and temporal or intervention-based observations that would favor, weaken, or redirect the proposed causal ordering.

The reported findings and proposed mechanistic bridges are not assigned the same evidentiary status. The organizing question is whether, under the M1-primary starting hypothesis, known forms of neural plasticity could plausibly contribute to the observed extra-M1 phenotype. Compatibility with that sequence is not treated as evidence that its causal direction has been established.

### 2.1. Basal Ganglia: Dopaminergic and Pathway Reweighting

The empirical starting point is a set of task-linked striatal abnormalities reported in FTSD. PET studies in writer’s cramp and laryngeal dystonia have identified increased striatal D1-receptor availability, reduced D2/D3-receptor availability, task-dependent alterations in dopamine release, and diminished spatial overlap among these measures [4,15,16]. These observations establish striatal involvement but do not directly measure receptor expression, synaptic efficacy, direct- or indirect-pathway output, or causal ordering. The same findings could reflect an initiating basal ganglia abnormality, an adaptation to repeated dystonic output, a compensatory response, or a concurrent component of distributed pathology.

Within the proposed M1-primary sequence, repeated recruitment of the dystonic synergy within the affected M1 TSMS is hypothesized to engage a corresponding task-related basal ganglia channel. Midbrain dopamine neurons receive convergent input from motor, somatosensory, basal ganglia, and brainstem structures, including the striatum, globus pallidus, subthalamic nucleus (STN), and pedunculopontine nucleus (PPN) [17,18,19]. Direct- and indirect-pathway striatal projection neurons are intermingled and receive overlapping classes of cortical, thalamic, and dopaminergic input, although the relative weighting of particular inputs can differ between pathways [20]. This organization provides a substrate for task- or channel-biased functional reweighting without requiring anatomically separate direct- and indirect-pathway territories.

At the dopamine-neuron level, phasic recruitment depends on coordinated excitatory input, inhibitory gating, and the intrinsic propensity of substantia nigra pars compacta (SNc) neurons to transition from tonic firing to burst firing [21,22,23,24,25]. We hypothesize that repeated abnormal output and error-related sensory consequences during the affected task could alter the timing or gain of the convergent inputs controlling this transition, so that the same motor context becomes less likely to evoke a large phasic dopamine response. Activity-dependent modification of glutamatergic afferents, reweighting of excitatory and inhibitory inputs within the basal ganglia–brainstem loop, or homeostatic changes in burst-related intrinsic conductances are non-exclusive candidate routes [26,27,28]. The cited studies establish that these substrates are modifiable; they do not demonstrate that any one of them is altered in FTSD. The central proposal is, therefore, a context-dependent change in effective dopamine recruitment, not a required sequence involving a particular afferent, ion channel, or formal reward-prediction-error process.

At the striatal level, abnormal dopamine magnitude or timing, when repeatedly paired with persistent glutamatergic input generated during recruitment of the dystonic synergy within the affected M1 TSMS, could bias plasticity differently across direct- and indirect-pathway projection neurons. Direct-pathway neurons preferentially express D1 receptors, whereas indirect-pathway neurons preferentially express D2 receptors, and the two receptor classes engage different intracellular regulatory pathways [29,30]. The narrower hypothesis is that repeated abnormal cortical input and altered dopamine signaling could favor relative direct-pathway facilitation, reduce the effective capacity of indirect-pathway control, or produce both changes within the affected task channel. This does not require complete anatomical separation of the pathways and does not assume that PET receptor availability is a direct measurement of receptor trafficking, synaptic efficacy, or pathway activity.

Once established, such reweighting could become a reinforcing or maintaining mechanism even if it did not initiate FTSD. Greater facilitation of the affected task channel or less effective suppression of competing output could increase the probability that subsequent recruitment of that dystonic synergy produces abnormal movement. Alternatively, some of the measured striatal changes could oppose the cortical disturbance and represent incomplete compensation. The functional role of a PET abnormality, therefore, cannot be inferred solely from whether receptor availability or dopamine release is increased or reduced.

The existing cross-sectional imaging findings are compatible with this proposed route but are not discriminating evidence for M1 causal primacy. Stronger support would require longitudinal evidence that task-specific abnormalities of M1 recruitment or rapid inhibitory control precede or closely track the emergence of striatal changes within the corresponding task channel. During successful BATR or another effective intervention, improvement of the cortical phenotype should be followed by, or covary with, normalization of task-linked dopamine or receptor-availability measures and an independently measured increase in the symptom-threshold. Evidence that striatal abnormalities consistently precede M1 dysfunction and independently predict later symptom emergence, or that a selectively basal ganglia-directed intervention normalizes M1 physiology and behavior before detectable cortical change, would favor a basal ganglia-primary or bidirectional account. Persistence of a striatal abnormality after durable normalization of M1 physiology and task performance would weaken a simple reversible downstream interpretation, although persistence alone could not distinguish an irreversible downstream adaptation from an independent or upstream abnormality.

### 2.2. Cerebellum: Recalibration to Recurrent Error-Related Input

The empirical starting point is the reported presence of impaired cerebellar modulation of M1, altered cerebello-cortical connectivity, and task-related differences in cerebellar activity in focal dystonia [31,32,33]. These findings establish cerebellar involvement but do not determine whether the abnormality initiates the disorder, develops in parallel with M1 dysfunction, adapts to repeated dystonic output, or compensates for it.

Within the proposed M1-primary sequence, repeated expression of the dystonic synergy within the affected M1 TSMS is expected to generate recurring discrepancies among the intended motor command, realized movement, and resulting sensory feedback. We hypothesize that repeated exposure to these abnormal task-bound consequences could progressively alter the relationship between error-related input and cerebellar corrective output. This proposal does not require every dystonic contraction to constitute a formal teaching signal or assume that the cerebellum is simply exhausted. It asks whether persistent, poorly resolved, or repeatedly similar error-related input could make subsequent cerebellar updating less selective or less proportional to the immediate deviation.

Climbing-fiber input to Purkinje cells provides one plausible entry point. In established models of cerebellar learning, as reviewed by Yamazaki and Lennon [34], climbing-fiber activity generates large dendritic calcium signals and helps instruct plasticity at parallel fiber–Purkinje cell synapses; the size and temporal organization of climbing-fiber events can influence the direction and magnitude of learning [35,36,37]. Climbing-fiber synapses, Purkinje-cell intrinsic excitability, and the timing of olivocerebellar activity are themselves modifiable [38,39,40,41,42]. Reported changes in Purkinje-cell intrinsic excitability following synaptic plasticity differ in direction across induction conditions [43,44]. Recurrent, diffuse, or poorly timed recruitment of these systems could, therefore, reduce the temporal selectivity or effective dynamic range of later instructive events. These studies establish the plasticity of the relevant substrates; they do not demonstrate that climbing-fiber efficacy, inferior-olive coupling, HCN1 function, or any particular Purkinje-cell conductance is altered in human FTSD.

Changes in Purkinje-cell activity could subsequently reshape deep cerebellar nuclear (DCN) output. Purkinje synchrony influences the timing of cerebellar nuclear spiking, and Purkinje-to-DCN inhibitory synapses can undergo activity-dependent gain changes [45,46,47]. We, therefore, propose that repeated abnormal task-related input could recalibrate cerebellar output around the dystonic motor state, making cerebello-thalamo-cortical feedback less effective at constraining abnormal M1 recruitment. In this usage, cerebellar recalibration denotes a hypothesized change in the mapping from error-related input to corrective output. It does not denote a directly measured cerebellar error threshold, complete loss of cerebellar function, or absence of ongoing corrective engagement.

This formulation allows the cerebellar abnormality to have different functional roles at different stages. Elevated cerebellar activity during dystonic performance could reflect persistent attempts to correct abnormal movement, maladaptive updating around repeatedly expressed dystonic output, or a mixture of both. Altered connectivity or impaired cerebellar modulation of M1 could likewise represent an incomplete compensatory response rather than a purely pathological change. Once established, however, a cerebellar adaptation could become reinforcing or maintaining by providing less effective constraint on M1, repeatedly returning altered corrective signals to the cortical network, or both. Thus, a cerebellar change hypothesized to be downstream during symptom formation could later become causally important for persistence.

The available human findings are compatible with this proposed route but do not discriminate it from cerebellar-primary, bidirectional, or concurrent accounts. A downstream sequence predicts that task-specific abnormalities of M1 recruitment or rapid inhibitory control will precede or closely track the emergence of altered cerebellar modulation, connectivity, or task-related activity. Cerebellar abnormalities should be strongest under conditions that recruit the affected TSMS and generate the relevant abnormal output. During successful BATR or another effective intervention, improvement in the cortical phenotype and an independently measured increase in the symptom-threshold should be followed by, or covary with, normalization of cerebellar modulation or task-linked cerebellar activity. Evidence that cerebellar abnormalities reproducibly precede M1 dysfunction and independently predict later symptom emergence, or that a selectively cerebellar-directed intervention restores behavior and M1 physiology before detectable cortical change, would favor a cerebellar-primary or bidirectional account. Simultaneous changes without a reproducible temporal leader would remain more consistent with a concurrent or distributed sequence.

### 2.3. Primary Somatosensory Cortex (S1): Altered Functional Sensory Separability as a Candidate Adaptation

The empirical literature on S1 organization in FTSD is mixed. Classical MEG- and EEG-based studies reported reduced separation or altered organization of digit-related responses in focal hand dystonia, including descriptions of an abnormal somatosensory homunculus or overlapping digit representations [48,49,50]. In a nonhuman-primate overuse model, repetitive hand use produced dedifferentiation of the S1 hand representation together with motor abnormalities resembling focal dystonia [51]. By contrast, high-resolution fMRI found that generic static finger representations in primary sensorimotor cortex could remain intact in musician’s dystonia [52]. These findings do not support a universal claim that static digit maps are anatomically merged in FTSD.

Other human studies have reported abnormal central integration of paired somatosensory inputs, impaired paired-pulse somatosensory inhibition, abnormal temporal discrimination, and disordered associative plasticity in S1 [53,54,55,56]. Static spatial maps, temporal separation of evoked responses, task-dependent interactions, and inhibitory gating are related but non-equivalent properties. We, therefore, use altered functional sensory separability to denote a possible task- or state-dependent reduction in temporal separation, inhibitory isolation, or functional differentiation among neighboring sensory populations, not a universally present anatomical fusion of digit maps.

Functional separation can depend on active inhibitory gating even when cortical territories overlap. Neurons assigned to one digit representation in primate area 3b can respond to other digits and can be modulated by concurrent cross-digit input [57]. Neocortical inhibitory circuitry is organized principally through PV-, SST-, and 5HT3aR-related families [58,59]. Thalamocortical input strongly recruits fast-spiking interneurons and generates rapid feedforward inhibition that narrows the temporal integration window of principal neurons, while SST- and 5HT3aR-related populations contribute delayed, dendritic, state-dependent, and disinhibitory control [60,61,62,63,64,65,66]. These findings make rapid PV-centered inhibitory sharpening one plausible contributor to sensory separability, but cell-type-resolved evidence has not established a PV-specific S1 abnormality in human FTSD.

Within the proposed M1-primary sequence, repeated expression of the dystonic synergy within the affected M1 TSMS could alter the statistics of sensory input returning to S1. During the affected task, mechanical enslaving, motor overflow, or simultaneous recruitment of neighboring effectors can produce correlated movement and afferent input across adjacent digits [67,68,69]. Adult-primate studies show that experimentally increasing temporal coupling between adjacent digits can reorganize area 3b, whereas behaviorally controlled tactile experience can reshape representations according to patterns of use and timing [70,71,72,73]. We hypothesize that recurrent dystonic coactivation could similarly strengthen or unmask cross-digit interactions and reduce the effectiveness of inhibitory timing relationships that normally decorrelate neighboring populations. The predicted change is task-linked functional coupling, not complete anatomical fusion.

At the synaptic level, the responsible locus is unknown. Strengthened horizontal excitation, unmasking of pre-existing cross-digit responses, mistimed or insufficient local inhibition, altered top-down modulation, and activity-dependent changes in inhibitory receptor organization are non-exclusive possibilities. Studies outside human FTSD show that increased excitatory activity and NMDA-receptor activation can modify GABAA-receptor clustering and mobility and that inhibitory LTD can involve calcineurin-dependent regulation of GABAA-receptor γ2 subunits [74,75,76]. These findings establish that inhibitory synaptic organization is activity-dependent; they do not demonstrate receptor redistribution in S1 in FTSD. The narrower hypothesis is that repeated task-linked cross-digit input progressively shifts the effective balance between cross-digit excitation and inhibitory sharpening toward more correlated sensory processing.

An S1 abnormality could acquire different roles over time. Reduced sensory separability could reinforce or help maintain dystonic output by degrading task-relevant sensory distinctions and returning less differentiated feedback to motor circuits. Alternatively, increased sensory recruitment could represent an incomplete compensatory attempt to monitor abnormal movement. Sensory dysfunction could also precede M1 abnormalities, contribute concurrently to maladaptive learning, or interact bidirectionally with the motor disturbance. The downstream-adaptation hypothesis, therefore, applies most directly to FTSD phenotypes in which task-related coactivation and a corresponding sensory abnormality can both be demonstrated.

Under a downstream sequence, task-specific abnormalities of M1 recruitment or rapid inhibitory control and the emergence of abnormal coactivation should precede or closely track changes in S1 inhibition, temporal discrimination, or task-related digit separability. The sensory abnormality should be most evident under conditions that recruit the affected TSMS and generate correlated afferent input. During successful BATR or another effective intervention, reduced dystonic coactivation and improvement in M1 motor selectivity should be followed by, or covary with, improved sensory separability and an independently measured increase in the symptom-threshold. Reports of sensory reorganization after behavioral treatment are compatible with this possibility [13,77], but BATR also changes sensory experience directly, so S1 normalization during retraining would not by itself establish M1-to-S1 causation. Evidence that S1 dysfunction consistently precedes M1 abnormalities and symptoms, independently predicts later onset, or that a selectively sensory-directed intervention raises the symptom-threshold and normalizes M1 physiology before detectable direct cortical change would favor a sensory-primary or bidirectional account.

### 2.4. Spinal Circuits: Reduced Reciprocal Inhibition as a Candidate Use-Dependent Adaptation

The empirical starting point is reduced reciprocal inhibition between forearm antagonists in focal hand dystonia, particularly writer’s cramp. Attenuation has been reported in several phases of reciprocal inhibition, although the affected phase has varied across studies and abnormalities have also been detected in clinically asymptomatic limbs [78,79,80]. These observations establish altered spinal inhibitory physiology but do not determine whether it initiates the disorder, develops in parallel with abnormalities elsewhere in the motor network, adapts to repeated dystonic output, or reflects compensation.

Reciprocal inhibition is dynamically regulated rather than fixed. Its expression varies with task demands, contraction state, co-contraction, descending corticospinal input, and peripheral afference, and spinal inhibitory interneurons contribute directly to appropriately timed antagonist relaxation [81,82,83,84,85,86]. It is also modifiable: patterned sensory stimulation, motor-skill learning, peripheral input, and brief exercise can alter reciprocal inhibition or the responsiveness of spinal inhibitory pathways [87,88,89,90]. Priori et al. [91] further reported that clinical improvement after botulinum toxin treatment was accompanied by increased reciprocal inhibition. These findings demonstrate physiological plasticity but do not establish that dystonic co-contraction causes a persistent weakening of the pathway.

Within the proposed M1-primary sequence, repeated recruitment of the dystonic synergy within the affected M1 TSMS could supply the abnormal activity pattern that drives spinal adaptation. Flexion- and extension-related M1 populations are partly overlapping, and disturbed surround inhibition in focal hand dystonia may permit descending recruitment to spread beyond the intended directional output [68,69,92]. A predominantly flexion- or extension-related dystonic state could, therefore, deliver overlapping drive to agonist and antagonist motoneuron pools. Co-contraction is not a uniquely cortical signature, however, and may also be influenced by basal ganglia, cerebellar, brainstem, sensory, peripheral, or spinal abnormalities.

We hypothesize that repeated overlapping agonist–antagonist drive could progressively alter the effective gain, recruitment, or descending regulation of reciprocal-inhibition pathways. A transient reduction in antagonist suppression during co-contraction should be distinguished from a durable use-dependent adaptation. The proposed longer-term change could involve altered transmission from group I afferents, altered corticospinal recruitment of inhibitory interneurons, or reweighting within spinal premotor inhibitory circuitry. Spike-timing-dependent plasticity demonstrated in other excitatory and inhibitory systems provides a general analogy for activity-dependent reweighting but does not identify the learning rule operating in human reciprocal-inhibition pathways in FTSD [93,94,95]. The narrower hypothesis is, therefore, that repeated abnormal use reduces effective reciprocal inhibition within the affected motor context without assigning the change to one required synapse or cellular mechanism.

Reciprocal inhibition should not be treated as interchangeable with the cortical silent period (CSP) or long-interval intracortical inhibition (LICI). The earlier portion of the CSP can include spinal contributions, whereas later portions depend more strongly on reduced motor-cortical excitability [96,97,98]. CSP shortening has been reported across isolated dystonias, but LICI findings in FTSD are heterogeneous, including normal, reduced, and increased inhibition [99,100,101,102,103]. A shortened CSP, therefore, cannot be attributed predominantly to reduced reciprocal inhibition, and neither measure uniquely identifies a cortical or spinal causal mechanism. Simultaneous measurement of reciprocal inhibition, peripheral or cervicomedullary indices, CSP duration, and LICI would be required to determine whether these abnormalities covary or dissociate during symptom expression and recovery.

Once established, reduced reciprocal inhibition could become reinforcing or maintaining even if it did not initiate FTSD. Less effective antagonist relaxation would favor repeated co-contraction during subsequent recruitment of the affected movement, allowing each above-threshold attempt to reproduce both the abnormal descending command and spinal conditions that stabilize the motor pattern. Alternatively, some measured spinal changes could reflect altered peripheral afference, task strategy, or incomplete compensation rather than persistent synaptic weakening. Abnormalities in asymptomatic limbs may likewise indicate a broader physiological trait rather than a purely task-acquired consequence. The direction of a reciprocal-inhibition change, therefore, does not by itself establish its functional or causal role.

Under a downstream sequence, task-specific abnormalities of M1 recruitment or rapid inhibitory control and the emergence of abnormal agonist–antagonist coactivation should precede or closely track reduced reciprocal inhibition. The spinal abnormality should be most evident under conditions that recruit the affected TSMS and reproduce the relevant co-contraction pattern. During successful BATR or another effective intervention, reduced dystonic coactivation and recovery of cortical motor selectivity should be followed by, or covary with, improved reciprocal inhibition and an independently measured increase in the symptom-threshold. Because botulinum toxin can alter both motor output and peripheral afference, spinal improvement during treatment would not by itself demonstrate correction of an M1-initiated mechanism. Evidence that spinal inhibitory abnormalities consistently precede M1 dysfunction and independently predict later symptom emergence, or that a selectively spinal- or peripheral-directed intervention restores M1 physiology and behavior before detectable cortical change, would favor a spinal-primary or bidirectional account. Persistence of reduced reciprocal inhibition after durable normalization of M1 physiology and task performance would weaken a simple reversible downstream interpretation, although it could still reflect a stable downstream adaptation.

## 3. Discussion

### 3.1. A Causal-Architecture Taxonomy of Dystonia and Its Implications for Motor Retraining

The clinical label “dystonia” encompasses disorders whose similar outward phenomenology, including patterned contraction, twisting, tremor, or abnormal posturing, may conceal substantially different initiating mechanisms and pathophysiological trajectories [104,105]. Phenomenological similarity, therefore, does not establish etiological equivalence, and findings obtained in one dystonia subtype should not automatically be generalized to another. The taxonomy proposed here is a hypothesis-level interpretive framework layered onto existing clinical classifications rather than a replacement for them or an individual-level diagnostic algorithm [1].

The classification axis is the hypothesized dominant causal architecture. In this usage, primary and dominant are related but not synonymous. Primary refers to causal initiation, whereas dominant refers to the process or interaction hypothesized to exert the greatest continuing causal constraint on generation and persistence of the core dystonic phenotype. A dominant mechanism need not be the only mechanism, and its relative importance may change across disease stages as secondary adaptations become reinforcing, maintaining, or compensatory. Classification should, therefore, be based on the best-supported causal sequence and continuing disease mechanism rather than on the most conspicuous physiological abnormality at a single time point.

We retain three category labels as mechanistic shorthand. In typical neuroplastic dystonia, maladaptive remodeling of motor-system circuitry is hypothesized to be the dominant proximate driver of the dystonic phenotype. In atypical neuroplastic dystonia, a defined genetic, molecular, biochemical, or structural abnormality exerts a stronger causal constraint, while compensatory and maladaptive plasticity substantially shapes whether, when, and how the phenotype is expressed. In non-neuroplastic dystonia, a persistent biochemical, metabolic, degenerative, genetic, or structural pathology is hypothesized to dominate both the underlying disease process and continuing symptom generation, while plasticity has a secondary, modifying, or compensatory role. “Typical” and “atypical” do not refer to prevalence, severity, or clinical normality, and “non-neuroplastic” does not imply that neural adaptation is absent.

**Figure 1:**
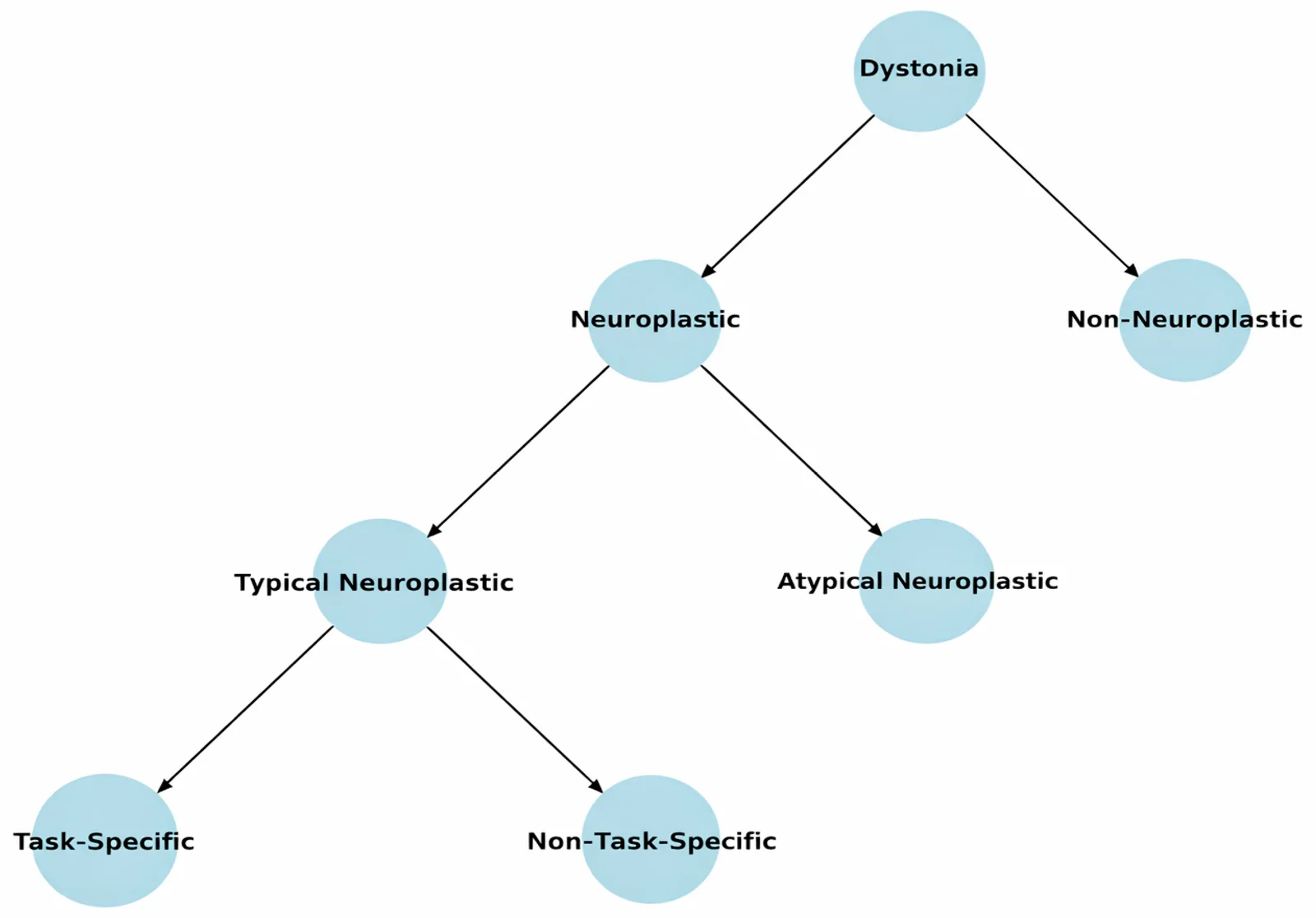
Proposed causal-architecture taxonomy of dystonia. The three principal categories, typical neuroplastic, atypical neuroplastic, and non-neuroplastic, represent hypothesized dominant causal architectures rather than mutually exclusive biological ingredients or an individual-level diagnostic algorithm. In typical neuroplastic dystonia, maladaptive motor-system plasticity is proposed to be the dominant proximate driver. The “Non-Task-Specific” branch represents candidate non-task-specific forms whose placement within typical neuroplastic dystonia remains unresolved pending convergent mechanistic evidence. Its inclusion does not imply automatic assignment of cervical dystonia, blepharospasm, or oromandibular dystonia to this category. In atypical neuroplastic dystonia, a stronger genetic, molecular, biochemical, or structural disturbance interacts with compensatory or maladaptive plasticity. In non-neuroplastic dystonia, a persistent biochemical, metabolic, degenerative, genetic, or structural pathology is proposed to dominate the underlying disease process and continuing symptom generation. “Typical” and “atypical” are mechanistic labels rather than statements about prevalence or severity, and “non-neuroplastic” does not imply an absence of neural adaptation. Mixed, boundary, evolving, and unresolved cases may remain provisionally unassigned.

These categories describe proposed dominant causal architectures rather than mutually exclusive biological ingredients or quantitative proportions of “genetic” and “plastic” influence within an individual. Genetic susceptibility, environmental exposure, learning, compensation, and network adaptation may contribute across all three categories. The FTSD-specific sequence involving a TSMS, symptom-threshold, proposed true-weakness state, overreaching, and BATR is not used as a universal sorting rule for all dystonias. Cases with mixed, boundary, evolving, or unresolved etiologies may remain provisionally unassigned until their causal architecture is better established. The proposed organization is summarized schematically in Figure 1.

Task-specific FTSD phenotypes provide the clearest candidate examples of typical neuroplastic dystonia. In musician’s dystonia and laryngeal dystonia, symptoms are linked to a particular learned motor context, lower-demand or symptom-sparing performance may remain possible, and a task-specific symptom-threshold may be measurable [11,13,106,107]. Reduced short-interval intracortical inhibition (SICI) and disturbed motor selectivity are consistent with a fast inhibitory phenotype in M1 [100,108,109,110,111,112]. These findings motivate a neuroplastic classification but do not by themselves identify a TSMS, establish PV-specific dysfunction, or demonstrate that maladaptive M1 plasticity initiated the disorder.

The more specific sequence imported from the companion framework, altered movement coordination, reduced functional TSMS capacity, a proposed state of task-specific output limitation termed true weakness, repeated overreaching, excitatory-biased plasticity, and formation of a threshold-dependent dystonic synergy, is proposed for FTSD rather than used as a universal sorting rule for the typical neuroplastic category [14]. Its strongest application is to phenotypes in which an affected task, an intact lower-demand range, and an individualized demand boundary can be identified reproducibly. Other neuroplastic dystonias could arise through different initiating events or plasticity sequences while still sharing the broader property that maladaptive motor-system remodeling is the dominant proximate driver.

Non-task-specific focal or segmental dystonias, including oromandibular dystonia, blepharospasm, and cervical dystonia, should not be assigned automatically to the typical neuroplastic branch solely because abnormalities of cortical excitability or inhibition have been reported [103,113,114,115]. A similar SICI phenotype does not establish the same initiating mechanism, and the symptom-threshold, overreaching process, and task-bound TSMS proposed for FTSD may not be identifiable. Classification of a non-task-specific phenotype as typical neuroplastic would require convergent evidence for a reproducible affected motor state, a plasticity-dependent circuit abnormality, and a relationship between modification of that abnormality and symptom change. Until such evidence is available, these phenotypes should be treated as candidate or unresolved cases rather than presumed extensions of FTSD.

Genetic susceptibility does not independently determine category membership. Non-manifesting DYT1 mutation carriers can display abnormalities of cortical inhibition without overt dystonia, indicating that a physiological predisposition may be present without being sufficient for clinical expression [116]. This observation illustrates that genetic susceptibility and maladaptive plasticity can coexist, but it does not establish that environmental overreaching is required in DYT1-associated dystonia or place all DYT1 phenotypes within the typical neuroplastic branch. A genetic variant could function as a susceptibility modifier, a strong initiating disturbance whose expression is shaped by adaptation, or the dominant continuing cause of the disorder.

Lesion-associated dystonia is an important boundary case because the structural injury is an established upstream event, whereas the mechanisms linking that injury to the later dystonic phenotype may vary substantially across patients [117]. Post-lesional plasticity, compensation, altered motor use, and behavioral experience can shape recovery and symptom expression [118,119]. In some cases, secondary maladaptive reorganization may become a major continuing driver; in others, the direct consequences of the lesion may remain dominant. Repeated attempts to exceed the capacity of residual motor circuitry could conceivably contribute to maladaptive reorganization in a subset, but an FTSD-like overreaching mechanism should not be assumed without independent evidence. Classification should, therefore, depend on the demonstrated relationship among the lesion, subsequent plasticity, and continuing symptom generation rather than on the presence of a lesion alone.

Atypical neuroplastic dystonias are proposed as disorders in which a defined genetic, molecular, biochemical, or structural abnormality exerts a strong causal constraint while compensatory and maladaptive plasticity substantially shapes whether, when, and how the phenotype becomes manifest. Incomplete penetrance, delayed onset, trigger sensitivity, or a period of preserved function may be compatible with this architecture but do not prove that compensatory failure is responsible. Rapid-onset dystonia-parkinsonism provides an illustrative candidate: ATP1A3 mutations constitute a defined molecular driver, while clinical onset may follow a period of preserved function and may occur in association with physiological or environmental stressors [120,121,122]. Support for an atypical neuroplastic classification would require evidence that adaptive network mechanisms materially delay, limit, or shape expression of the persistent molecular disturbance and that modifying those mechanisms changes the phenotype.

The non-neuroplastic category includes disorders in which a persistent biochemical, metabolic, degenerative, genetic, or structural pathology is hypothesized to dominate both the underlying disease process and continuing symptom generation. Candidate examples include dopa-responsive dystonia and X-linked dystonia-parkinsonism, in which disturbed dopamine synthesis or progressive basal ganglia pathology provides a comparatively direct disease mechanism [123,124,125]. Neural compensation, learning, and secondary network adaptation may still influence penetrance, severity, and functional outcome. The category, therefore, does not imply an absence of plasticity; it predicts that correction of maladaptive plasticity alone would not remove the persistent pathology that principally generates the disorder.

Across all three branches, no single feature, including mutation status, reduced SICI, delayed onset, exposure to a trigger, incomplete penetrance, or response to one intervention, is sufficient as an independent sorting rule. Classification should integrate the best available evidence concerning causal timing, the process on which continuing symptom expression depends, and the sequence of physiological and behavioral change after a selectively targeted intervention. Mixed architectures are expected, and the dominant mechanism may change across disease stages. When the relevant causal relationships remain unresolved, the disorder or individual case should remain provisionally unassigned rather than being classified under the dystonia label or one associated biomarker.

The therapeutic implications of the causal-architecture taxonomy are testable predictions rather than treatment rules assigned solely by diagnostic label. If maladaptive motor plasticity is the dominant driver of a disorder, appropriately structured retraining may have the potential to modify a causal circuit rather than only compensate for its consequences. If a persistent biochemical, molecular, structural, or degenerative pathology remains dominant, rehabilitation may still improve function, reduce secondary maladaptation, and strengthen compensation, but would not be expected to remove the primary pathology. The taxonomy, therefore, predicts the mechanistic role retraining might play; it does not divide patients into those who should or should not receive rehabilitation.

Task-specific FTSD provides the most direct setting in which to evaluate BATR. BATR should be understood as a structured, threshold-referenced form of symptom-sparing motor practice rather than as a wholly separate class of exercise. Generic slow practice may enter the BATR range when reducing speed places performance below the relevant boundary, but speed alone does not define BATR. Depending on the phenotype, the controlling task-demand variable may instead be force, pressure, amplitude, loudness, intereffector coordination, or a prespecified multidimensional index. What distinguishes BATR is that practice is defined relative to an individualized performance boundary, requires accurate translation of attempted demand into realized output, separates formal threshold assessment from routine practice, and uses prespecified monitoring, progression, and reassessment procedures. Slow-down exercise and related sensorimotor retraining provide clinical precedent for symptom-sparing practice but do not establish BATR efficacy or its proposed TSMS-level mechanism [11,12,13,14].

A minimally reproducible BATR protocol should prespecify the affected task and governing demand variable, estimate the symptom-threshold and, where present, the true-weakness threshold during a separate validation phase, and set practice conservatively at or below the lower relevant boundary. Routine practice should remain accurate and symptom-free, avoid deliberate threshold searching, return to the last stable intensity if dystonia, task-specific underproduction, disproportionate effort, compensatory movement, pain, or excessive fatigue occurs, and use prespecified progression and reassessment procedures. Dose, adherence, adverse effects, and task-performance change should be recorded; the companion article provides the fuller threshold-estimation, reliability, and progression procedures [14].

BATR efficacy and its proposed TSMS-level mechanism are separate empirical questions. Section 3.3.2 specifies the behavioral and physiological findings that would support or challenge each.

The task-specific BATR procedure should not be transferred unchanged to non-task-specific dystonias. An adapted threshold-referenced retraining approach would first require a reproducible affected motor state, triggering condition, or performance boundary; a prespecified variable governing that state and criteria distinguishing intended movement from compensation. Reduced SICI alone would be insufficient to establish that the FTSD-specific TSMS mechanism is operating. The relevant practice range, dose, progression rule, behavioral endpoint, and physiological target would need to be established independently for each phenotype. Use of the term BATR outside FTSD should, therefore, remain provisional unless an analogous task- or state-dependent performance boundary can be operationalized.

For atypical neuroplastic dystonias, retraining is predicted to have a more variable and generally adjunctive role, depending on residual compensatory capacity, persistence of the underlying disturbance, and availability of a modifiable motor pattern. For non-neuroplastic dystonias, treatment of the dominant pathology should remain mechanistically central; L-DOPA in dopa-responsive dystonia and pallidal deep brain stimulation in selected patients with X-linked dystonia-parkinsonism illustrate interventions directed more closely toward the relevant biochemical or circuit pathology [126,127]. These differential treatment predictions are formalized in Section 3.3.5.

### 3.2. Evidentiary Status, Limitations, and Competing Causal Sequences

The empirical foundation of this Hypothesis article consists of reported abnormalities in M1 physiology, striatal dopaminergic signaling, cerebellar activity and modulation, somatosensory processing, and spinal reciprocal inhibition in FTSD and related dystonias. The central unresolved question is the temporal and causal relationship among those abnormalities. Existing cross-sectional TMS, PET, functional-imaging, sensory, and spinal measurements can establish association, task dependence, and physiological covariation, but generally cannot determine whether a finding is initiating, maintaining, reinforcing, compensatory, or concurrent. The present article asks whether some extra-M1 findings could arise through repeated expression of the dystonic synergy within an affected M1 TSMS; compatibility with that sequence is not treated as evidence that M1 causal primacy has been established.

Several central elements remain inferential. An FTSD-specific TSMS has not yet been identified using convergent functional criteria or followed longitudinally from normal performance through symptom emergence; Section 3.3.1 specifies the observations required to test the construct. Reduced SICI, disturbed surround inhibition, and related TMS findings support a measurable deficit in rapid intracortical inhibitory control, but they do not identify the responsible interneuron class, quantify E→I or I→E synaptic efficacy within a TSMS, or establish PV-specific dysfunction. The PV-centered account is, therefore, the proposed cellular mechanism, whereas a fast inhibitory deficit compatible with PV-mediated circuitry is the better-supported physiological phenotype.

The extra-M1 measurements have corresponding limitations. PET receptor availability does not directly measure receptor expression, trafficking, synaptic efficacy, or direct- and indirect-pathway output. Functional connectivity and BOLD activity do not establish causal direction or determine whether cerebellar recruitment is maladaptive, corrective, or compensatory. The S1 literature does not support universal anatomical merging of digit maps: preserved static representations can coexist with abnormalities of temporal separation, inhibitory gating, or task-dependent functional sensory separability [52]. Findings for LICI are heterogeneous, and reciprocal inhibition, the cortical silent period, and cortical GABAB-related inhibition should not be treated as interchangeable measures [102,103].

Evidentiary strength is not uniform across the article. The network-adaptation hypotheses are developed specifically for FTSD and become more tentative when extended to non-task-specific, lesion-associated, or molecularly defined dystonias. The causal-architecture taxonomy is provisional and may ultimately prove dimensional rather than categorical; mixed, evolving, and etiologically unresolved cases are expected [8,104,105,117]. The clinical evidence motivating BATR-related principles is also limited to individual cases and relatively small cohorts, with continuing concerns about bias, control conditions, generalizability, and durability [11,12,13,128,129]. Behavioral improvement would test BATR efficacy, whereas associated physiological change would separately test the proposed TSMS-level mechanism.

Alternative causal sequences remain viable. Basal ganglia, cerebellar, somatosensory, premotor or associative cortical, brainstem, or spinal dysfunction could precede and induce abnormal M1 physiology. A common upstream disturbance could affect several motor-network nodes concurrently, or the initiating sequence could differ among FTSD phenotypes. Later bidirectional reinforcement is also compatible with an initially M1-primary sequence: even if the relevant circuit imbalance first arises within an M1 TSMS, subsequent basal ganglia, cerebellar, sensory, or spinal adaptations could feed back onto M1 and become reinforcing, maintaining, or necessary for persistence. The M1-primary claim, therefore, concerns causal initiation rather than permanent anatomical or functional dominance.

For evaluation of the framework, we distinguish compatible, supportive, and challenging or falsifying evidence. Compatible evidence is an observation that can be reconciled with the proposed sequence but does not distinguish it from viable alternatives; most existing cross-sectional abnormalities fall into this category. Supportive evidence requires a predicted temporal, task-specific, or intervention-related relationship, such as abnormal M1 recruitment preceding the corresponding extra-M1 change or cortical recovery preceding its normalization. A particular claim would be challenged by reproducible evidence inconsistent with its required sequence under adequately powered and appropriately controlled conditions. The M1-primary claim would be weakened if an extra-M1 abnormality consistently preceded M1 dysfunction and independently predicted later symptoms, or if selective correction of that extra-M1 system restored M1 physiology and behavior before detectable cortical change. The network-propagation claim would be weakened if sustained experimental induction of the proposed M1 abnormality failed to produce the predicted task-linked adaptations. The TSMS construct and causal-architecture taxonomy would likewise require revision if they failed the operational and predictive tests specified below. No single negative result necessarily rejects the entire framework, but repeated failure of a central prediction would challenge the corresponding component rather than being absorbed as merely another compatible network state.

### 3.3. Operational Criteria, Testable Predictions, and Falsification Criteria

#### 3.3.1. Operational Criteria for Identifying and Testing a TSMS

A TSMS is proposed as a task-dependent functional ensemble rather than a sharply bounded anatomical module. Evidence for its existence would, therefore, require convergence across several prespecified functional criteria; task-related M1 activation alone would be insufficient. Investigators should first define the movement component and task context to be tested. A candidate TSMS should then show a reproducible multivariate M1 population pattern across repeated trials and sessions, a stable temporal relationship to preparation and execution of the specified movement component, and localization within the relevant M1 representation while permitting partial overlap with ensembles supporting other movements. Crucially, the pattern should distinguish the target task from control conditions matched as closely as feasible for effector, movement direction, amplitude, speed, force, kinematics, surface electromyography, effort, and sensory input. Cross-validated decoding or representational analyses should demonstrate that task identity or context explains ensemble recruitment beyond generic movement output. Temporal coherence in this criterion refers to reproducible recruitment timing and population organization, not an obligatory oscillatory rhythm.

In FTSD, evidence that a candidate ensemble constitutes the affected TSMS would require more than identification of task-specific activity. The ensemble should show a reproducible abnormality in recruitment, gain, spatial spread, temporal organization, or rapid inhibitory control during the affected task relative to lower-demand symptom-free trials, matched control tasks, and appropriate control participants. That abnormality should vary systematically with the task-demand variable governing symptom expression, becoming more evident as performance approaches or crosses the symptom-threshold, while comparable high-demand movements outside the affected task remain relatively spared. Including both symptom-free and symptom-provoking conditions within the affected task would help distinguish effects of task identity, demand, and overt dystonic output. Task-specific changes in SICI or surround inhibition could support a fast-inhibitory phenotype but would not identify the ensemble’s cellular composition or establish PV specificity.

Stronger causal evidence would require selective perturbation or modification. In an experimental model, perturbing the candidate ensemble should preferentially alter the specified movement component rather than globally impair the effector, and manipulating rapid inhibitory control within that ensemble should shift the task demand at which abnormal output emerges. In humans, convergent evidence could come from task-specific stimulation or retraining combined with repeated population-level measurements, or from intracortical recordings where available for clinical reasons. Because current noninvasive human methods do not directly resolve a local E/I microcircuit, no single modality would be sufficient. The strongest intervention result would be a change in recruitment or inhibitory physiology within the same candidate ensemble whose magnitude predicts an independently measured shift in the symptom-threshold.

Failure to identify a reproducible task-specific M1 population pattern after adequately powered and appropriately controlled studies would argue against the TSMS as defined here. The construct would also be challenged if the apparent pattern were fully explained by generic kinematics, electromyographic activity, effort, or sensory input; if candidate-ensemble recruitment showed no reproducible relationship to symptom expression or the symptom-threshold; or if selective perturbation of the proposed ensemble failed to preferentially affect the specified movement component.

#### 3.3.2. Longitudinal Human Tests of Causal Ordering and BATR

Prospective longitudinal studies should combine prespecified task-specific behavioral testing with repeated measurements of M1 and extra-M1 physiology during symptom emergence, progression, and recovery. Behavioral assessment should quantify the symptom-threshold and independently measure attempted and realized output using task-appropriate kinematics, surface electromyography, and blinded clinical ratings, allowing intact performance, the proposed true-weakness state, compensatory movement, and overt dystonia to be distinguished. Physiological assessment should track candidate-TSMS recruitment and rapid inhibitory control in M1 together, where feasible, with task-linked striatal, cerebellar, somatosensory, and spinal measures [4,16,55,56,79,100,110,112].

The M1-primary sequence predicts that a reproducible task-specific abnormality of candidate-TSMS recruitment or rapid inhibitory control will be the earliest identifiable circuit-level change and will precede or closely track the corresponding extra-M1 abnormalities. The relevant extra-M1 changes should occur within the task-related or somatotopic channel engaged by the affected movement rather than appearing only as nonspecific whole-network differences. A basal ganglia, cerebellar, somatosensory, brainstem, or spinal abnormality that consistently precedes M1 dysfunction and independently predicts later symptom emergence would instead favor the corresponding extra-M1-primary or upstream account. Simultaneous changes across several nodes without a reproducible temporal leader would favor a concurrent or distributed sequence, whereas variation in the temporal leader among well-characterized participants would indicate etiological heterogeneity within FTSD.

BATR provides a complementary within-participant test during recovery. Clinical efficacy would be supported by an increase in the symptom-threshold or, where present, the true-weakness threshold that exceeds the relevant measurement-error or participant-specific uncertainty margin and is accompanied by improved accurate task performance. If improvement occurs through the proposed TSMS-level mechanism, recovery of candidate-ensemble recruitment, rapid intracortical inhibition, surround inhibition, or related measures of motor selectivity should precede or covary closely with the behavioral change. Later normalization of task-linked striatal, cerebellar, sensory, or spinal measures would support the proposed downstream sequence. Substantial behavioral improvement without corresponding recovery of M1 physiology would challenge the proposed TSMS-level mechanism without necessarily invalidating BATR as a rehabilitation strategy. Conversely, normalization of M1 physiology without a meaningful behavioral threshold shift would challenge the sufficiency of cortical rebalancing. Repeated failure to identify a true-weakness interval in adequately characterized cohorts would limit the generality of that construct rather than necessarily rejecting the remainder of the framework.

#### 3.3.3. Intervention-Based Discrimination Among Causal Models

Intervention studies provide stronger tests of causal ordering than physiological covariation alone, provided that target engagement is verified and measurements are obtained at sufficient temporal resolution. Under an M1-primary sequence, an intervention that preferentially restores task-specific M1 recruitment or rapid inhibitory control should first alter the cortical phenotype and raise the symptom threshold; normalization of associated extra-M1 abnormalities should follow or covary with that cortical change. The strongest support would come from reproducible within-participant ordering rather than from a single pre–post association.

The competing accounts make different directional predictions. A basal ganglia-primary sequence predicts that verified correction of task-linked striatal signaling or pathway function will precede restoration of M1 physiology and behavior. A cerebellar-primary sequence predicts that correction of abnormal cerebellar modulation or task-related output will precede cortical normalization. Cerebellar theta-burst stimulation has been used to probe arm and neck movement kinematics in focal dystonia, providing an example of a cerebellar-directed intervention paradigm without establishing cerebellar causal primacy by itself [130]. A sensory-primary sequence predicts that selective improvement of task-relevant sensory inhibition, temporal discrimination, or functional separability will precede improvement in M1 recruitment and the symptom-threshold. A spinal-primary sequence predicts that correction of the relevant spinal inhibitory abnormality will precede normalization of cortical and behavioral measures. A concurrent or distributed account predicts either no reproducible single temporal leader or a requirement for coordinated modification of more than one node before durable improvement occurs. Bidirectional models predict that correction at either of two reciprocally coupled nodes may initiate improvement, but that durable recovery will depend on modification of both.

These comparisons require caution because nominally selective interventions can influence connected regions. Botulinum toxin changes motor output and peripheral afference, and noninvasive brain stimulation can affect nodes beyond the nominal target. Studies should, therefore, document local target engagement, include appropriate sham or active-control conditions, and avoid assuming that the first measured change occurred at the directly targeted synapse or cell population. Persistence of an extra-M1 abnormality after durable normalization of M1 physiology, task performance, and the symptom threshold would weaken a simple reversible downstream interpretation, although it could reflect a stable downstream adaptation, trait marker, or independent abnormality. More decisive evidence against an exclusively M1-primary account would be reproducible restoration of M1 physiology and durable behavioral recovery after selective correction of an extra-M1 abnormality without an earlier detectable cortical change.

#### 3.3.4. Experimental-Model Tests of Network Propagation

Experimental models are required to test whether repeated expression of an abnormal M1 state is sufficient to produce the proposed changes elsewhere in the motor system. A useful design would first establish stable performance of a learned motor task and identify a candidate task-specific M1 ensemble using criteria analogous to those in Section 3.3.1. Rapid inhibitory control could then be manipulated selectively within that ensemble so that abnormal output emerges preferentially at higher task demand while lower-demand and matched control movements remain relatively preserved. Cell-type-specific recording and manipulation should test whether disruption of PV-centered control produces the predicted threshold-dependent and spatially diffuse motor output more consistently than comparable disruption of SST- or VIP-centered pathways [58,92].

Longitudinal recordings should determine whether repeated recruitment of the experimentally induced M1 state produces task- or channel-specific changes in striatal dopamine recruitment and pathway physiology, cerebellar teaching and corrective output, S1 functional sensory separability, and spinal reciprocal inhibition. One control group should receive comparable movement practice, sensory exposure, and task demand without the imposed M1 inhibitory abnormality. Additional controls should separate effects of overt abnormal movement from effects of the altered cortical state itself and should test whether initiating a comparable disturbance in an extra-M1 structure can produce the cortical and behavioral phenotype through an alternative sequence.

The network-propagation hypothesis would receive strong support if the induced M1 abnormality reproducibly precedes the extra-M1 changes, if those changes are concentrated within the corresponding task or somatotopic channel, and if restoration of M1 inhibitory control prevents or reverses them. It would be weakened if sustained abnormal M1 output failed to produce the predicted adaptations, if the same changes arose equally in control animals without the cortical abnormality, or if an extra-M1 change consistently emerged first. System-level tests should take priority over commitment to one cellular route. Cell-resolved studies can subsequently evaluate non-exclusive candidate substrates, including SNc afferent or intrinsic burst-related plasticity, direct- and indirect-pathway reweighting, olivocerebellar and Purkinje-cell plasticity, local S1 excitation and inhibitory sharpening, and spinal premotor inhibitory circuitry. The framework predicts the broader network adaptations but does not require any one listed synaptic mechanism to be necessary.

#### 3.3.5. Tests of the Causal-Architecture Taxonomy

The causal-architecture taxonomy should be evaluated prospectively rather than by retrospectively assigning categories after treatment outcomes are known. Studies should prespecify the observations used to infer initiating and continuing causal mechanisms, account for clinical severity, disease duration, body distribution, medication exposure, and prior treatment, and permit mixed or unresolved cases to remain unassigned. The categories should not be operationalized as quantitative “genetic” and “plasticity” scores or inferred from one mutation, physiological measure, trigger, or treatment response.

Typical task-specific neuroplastic dystonias are predicted to show the clearest measurable performance boundaries and the strongest coupling between retraining-related behavioral improvement and normalization of the implicated motor physiology. A non-task-specific phenotype should show a comparable relationship only if a reproducible affected motor state and modifiable plasticity-dependent circuit abnormality can be identified. Atypical neuroplastic dystonias should show more variable or incomplete responses determined partly by residual compensatory capacity and persistence of the underlying disturbance. In non-neuroplastic dystonias, rehabilitation may improve function and secondary adaptation, but durable correction of the core disorder should depend more strongly on treatment of the dominant biochemical, molecular, structural, genetic, or degenerative pathology.

The taxonomy would be supported if the proposed branches can be assigned with reasonable mechanistic consistency and predict reproducible differences in causal ordering, physiological change, and treatment dependence beyond those predicted by existing clinical classifications. It would require revision if category assignment proves unreliable, if the branches do not predict differential outcomes after adjustment for relevant clinical variables, or if disorders assigned to different branches consistently share the same initiating circuit abnormality and respond through the same physiological mechanism. Failure to outperform a simpler dimensional or clinical classification would indicate that the taxonomy functions only as a descriptive heuristic rather than as a useful causal framework.

## 4. Conclusions

This companion Hypothesis article extends the M1-centered TSMS framework by proposing how repeated expression of the dystonic synergy within an affected M1 TSMS could reshape striatal, cerebellar, somatosensory, and spinal circuits. Candidate routes include task-linked reweighting of dopamine recruitment and striatal pathway function, recalibration of cerebellar teaching and corrective output, reduced task-dependent functional sensory separability in S1, and use-dependent reduction in spinal reciprocal inhibition. These mechanisms are conditional hypotheses constrained by reported FTSD findings and broader experimental neurophysiology; they have not been observed as a complete longitudinal sequence.

The M1-primary claim concerns circuit-level causal initiation rather than anatomical exclusivity or permanent functional dominance. Extra-M1 abnormalities could instead be upstream causes, concurrent components of distributed pathology, or compensatory responses, and changes that initially develop downstream could later become reinforcing, maintaining, or necessary for persistence. The central question is, therefore, whether task-specific abnormalities of M1 ensemble recruitment and rapid inhibitory control consistently precede or closely track the corresponding extra-M1 changes and whether selective correction of the cortical state produces the predicted temporal sequence of behavioral and network recovery.

The proposed causal-architecture taxonomy addresses the related but broader possibility that disorders sharing dystonic phenomenology differ in the process that dominates symptom generation and persistence. Typical neuroplastic, atypical neuroplastic, and non-neuroplastic dystonias are proposed as provisional mechanistic categories rather than mutually exclusive biological ingredients or individual-level diagnoses. The FTSD-specific TSMS, symptom-threshold, proposed true-weakness state, overreaching, and BATR mechanisms are not assumed to generalize unchanged across dystonias. BATR is predicted to be most directly testable in task-specific neuroplastic phenotypes with a reproducible affected task and measurable performance boundary, whereas retraining may have a more adjunctive or compensatory role when a persistent molecular, biochemical, structural, genetic, or degenerative pathology dominates.

The framework should be judged by discriminating predictions rather than by its ability to accommodate cross-sectional abnormalities. Reproducible evidence that an extra-M1 abnormality precedes M1 dysfunction and independently predicts symptom emergence, that selective correction of an extra-M1 system restores M1 physiology and behavior before detectable cortical change, that experimentally induced abnormal M1 output fails to produce the predicted task-linked network adaptations, or that the causal-architecture taxonomy does not predict differential causal ordering or treatment dependence would require revision of the corresponding claims. Conversely, longitudinal, intervention-based, and experimental-model confirmation of the predicted sequences would support, but would not by itself prove, the proposed causal architecture.

## Data Availability

The original contributions presented in this study are included in the article. Further inquiries can be directed to the corresponding author.
